# Prognostic Significance of SULF2 Expression in Surgically Resected Non-Small Cell Lung Cancer

**DOI:** 10.3390/medsci14020215

**Published:** 2026-04-26

**Authors:** Hakan Taban, Murat Ozdede, Orkun Akman, Sevgen Celik Onder, Saadettin Kılıckap

**Affiliations:** 1 Medical Oncology Clinic, Medical Park Ankara Hospital, Ankara 06680, Turkey; 2 Department of Internal Medicine, Faculty of Medicine, Hacettepe University, Ankara 06230, Turkey; mozdede@gmail.com; 3 Department of Pathology, Faculty of Medicine, Hacettepe University, Ankara 06230,Turkey; akman_orkun@hotmail.com (O.A.); sonder.hacettepe@gmail.com (S.C.O.); 4 Department of Medical Oncology, Faculty of Medicine, Istinye University, Istanbul 34010, Turkey; skilickap@yahoo.com

**Keywords:** biomarker, non-small cell lung cancer, prognosis, SULF2 expression, survival

## Abstract

Background: Sulfatase 2 (SULF2) is an extracellular enzyme involved in the modulation of multiple oncogenic signaling pathways and has been implicated in tumor progression across several malignancies. However, its prognostic significance in surgically resected non-small cell lung cancer (NSCLC) remains incompletely defined. Methods: SULF2 expression was evaluated by immunohistochemistry in tumor specimens from patients with stage I–III NSCLC who underwent curative-intent surgical resection between 2009 and 2016. Expression levels were quantified using an H-score-based system and categorized as low or high. Associations between SULF2 expression, clinicopathological characteristics, and survival outcomes, including overall survival (OS) and disease-free survival (DFS), were analyzed using Kaplan–Meier estimates and Cox proportional hazards models. Eighty-three patients were included, of whom 65 (78.3%) were male; 42.2% had stage I disease, 32.5% stage II, and 25.3% stage III. Results: SULF2 expression was detected in 94.0% of tumors, with 43 patients (51.8%) classified as high and 40 (48.2%) as low expression based on H-score. Overall survival and DFS did not differ significantly between SULF2-low and SULF2-high groups (log-rank *p* = 0.213 and *p* = 0.660, respectively). Median OS was 113.2 months (95% CI: 84.0–142.4) in the SULF2-low group and 54.9 months (95% CI: 39.4–186.9) in the SULF2-high group, while median DFS was 88.3 months (95% CI: 46.5–130.2) and 53.3 months, respectively, with numerically shorter survival in the SULF2-high group. Subgroup analyses stratified by pathological stage and histological subtype revealed no significant associations between SULF2 expression and survival outcomes. In multivariate analysis, SULF2 expression was not independently associated with either OS or DFS. Conclusions: SULF2 expression was highly prevalent in surgically resected NSCLC; although higher expression showed numerically poorer survival, this difference did not reach statistical significance.

## 1. Introduction

Lung cancer remains the leading cause of cancer-related morbidity and mortality worldwide, with nearly 2.5 million new cases and more than 1.8 million deaths reported in 2022 [1]. Lung cancer is traditionally classified into two major histological subtypes, small-cell lung cancer and non–small cell lung cancer (NSCLC), the latter accounting for approximately 85% of all cases, with adenocarcinoma (AC) and squamous cell carcinoma (SCC) representing the most common subtypes [2]. Despite the emergence of lung cancer screening programs in selected populations, more than half of patients are still diagnosed with locally advanced or metastatic disease [3].

For patients with resectable stage I–III NSCLC, surgery remains the cornerstone of curative-intent treatment [2,3]. In recent years, perioperative management has evolved substantially with the integration of targeted therapies and immune checkpoint inhibitors in addition to conventional chemotherapy [4]. Nevertheless, disease recurrence remains a major clinical challenge, and survival outcomes after surgery vary considerably. Current risk stratification relies largely on conventional clinicopathological parameters, and disease stage alone fails to fully capture the underlying biological heterogeneity that drives differences in recurrence risk and long-term survival [5]. This limitation highlights the need for additional prognostic biomarkers that more accurately reflect tumor biology and improve long-term survival stratification beyond conventional parameters.

Among the various molecular candidates emerging from tumor biology research, sulfatases have gained significant attention due to their critical regulatory roles in the tumor microenvironment [6]. Sulfatases constitute a large family of enzymes that catalyze the hydrolysis of sulfate esters and regulate diverse biological processes [7]. Among these, the extracellular sulfatases SULF1 and SULF2 are predominantly localized at the cell surface and modulate cellular signaling by altering the sulfation patterns of heparan sulfate proteoglycans [7,8]. Through these mechanisms, SULFs regulate ligand–receptor interactions involving multiple growth factors, cytokines, and morphogens, thereby influencing downstream signaling pathways critical for cell proliferation, differentiation, and migration [6,9,10].

Accumulating evidence suggests divergent roles for SULF1 and SULF2 in cancer biology [6]. While SULF1 is generally considered to exert tumor-suppressive effects, SULF2 has been implicated in pro-tumorigenic processes [6,8]. Increased SULF2 expression has been reported in several malignancies, including breast cancer, prostate cancer, gastric cancer, head and neck squamous cell carcinoma (HNSCC), hepatocellular carcinoma, bladder cancer, and adrenocortical carcinoma (ACC), primarily in translational and molecular studies evaluating tumor tissue expression levels [11,12,13,14,15,16,17]. In addition, elevated SULF2 expression has been associated with tumor progression and adverse survival outcomes in various malignancies, including hepatocellular carcinoma, HNSCC, pancreatic ductal adenocarcinoma, bladder cancer, and ACC [15,16,17,18,19]. These findings support a role for SULF2 as a modulator of oncogenic signaling and tumor–microenvironment interactions, providing a biological rationale for its evaluation as a potential prognostic biomarker.

In lung cancer, studies evaluating the expression and clinical relevance of SULF2 are limited. Initial evidence from experimental models demonstrated SULF2 expression in both lung AC and SCC and suggested a functional role in tumorigenesis through modulation of heparan sulfate sulfation and oncogenic signaling pathways, including *Wnt* signaling [20]. Subsequent clinical investigations reported frequent SULF2 expression in resected NSCLC specimens; however, its prognostic impact has been inconsistent and appears to vary by histological subtype [21]. Moreover, studies evaluating circulating SULF2 as a diagnostic biomarker have yielded heterogeneous results, partly influenced by patient-related factors such as age and comorbidities [21,22]. Collectively, these findings suggest that although SULF2 is biologically active in lung cancer, its definitive prognostic value in NSCLC remains incompletely defined and requires further clinical validation. Therefore, additional clinical studies are warranted to better clarify the prognostic significance of SULF2 in surgically resected NSCLC.

In this context, the present study aimed to investigate the prognostic significance of SULF2 expression in a retrospective cohort of patients with surgically resected stage I–III NSCLC. We evaluated the associations between SULF2 expression status, clinicopathological characteristics, overall survival, and disease-free survival to assess whether SULF2 may serve as a clinically relevant prognostic biomarker in NSCLC.

## 2. Materials and Methods

### 2.1. Study Design and Patient Population

**Figure 1:**
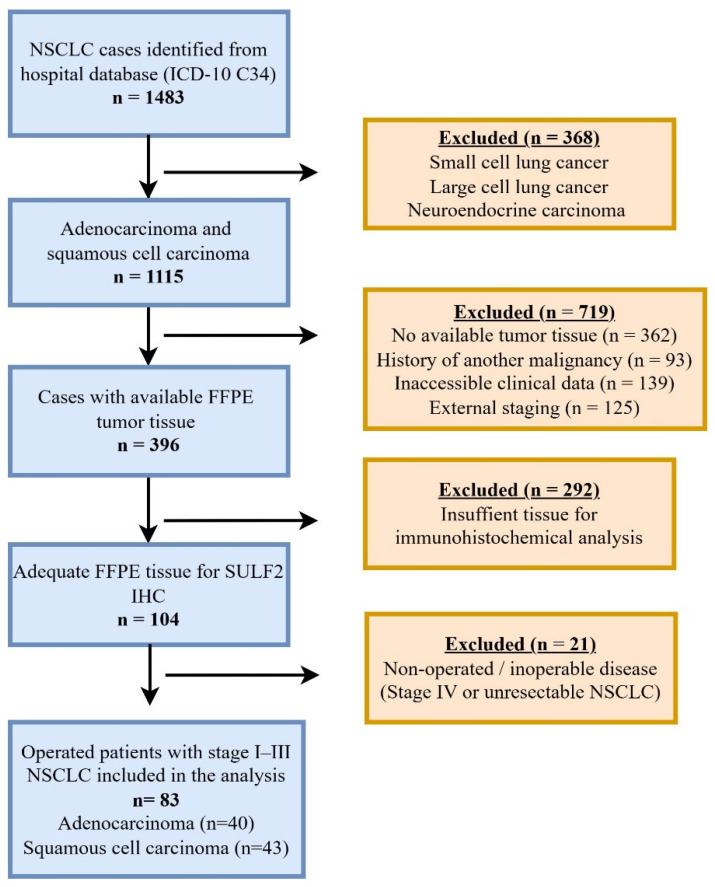
Flow diagram illustrating patient identification, exclusion criteria, and final study cohort selection. FFPE, formalin-fixed, paraffin-embedded; IHC, immunohistochemistry; NSCLC, non–small cell lung cancer.

This retrospective cohort study included patients diagnosed with NSCLC and treated at Hacettepe University Hospitals (HUH) between January 2009 and December 2016. Patients were identified through the hospital electronic database using the ICD-10 code C34. After applying predefined inclusion and exclusion criteria, including histological subtype, tissue availability, clinical data completeness, disease stage, and operability, only patients with AC or SCC who underwent curative-intent surgical resection for pathologically confirmed stage I–III disease were included. The final study population consisted of 83 patients who underwent surgical resection, including 40 with AC and 43 with SCC. The inclusion and exclusion criteria used for patient selection are summarized in Table 1, including definitions of clinical data completeness and tissue adequacy. A detailed flow diagram illustrating patient identification, exclusion criteria, and final cohort selection is shown in Figure 1.

The study protocol was approved by the Hacettepe University Non-Interventional Clinical Research Ethics Committee (Approval No: GO 17/192-10, dated 14 March 2017) and was conducted in accordance with the Declaration of Helsinki. Due to the retrospective nature of the study, the requirement for informed consent was waived. The dataset used in the present analysis was derived from the author’s previously completed master’s thesis entitled “Evaluation of SULF2 Expression in Diagnosis, Treatment Response, and Prognosis of Non-Small Cell Lung Cancer” completed at Hacettepe University.

### 2.2. Clinical and Pathologic Data

Demographic and clinical characteristics, including age, sex, smoking status (pack-years, current/former/never), comorbidities, ECOG performance status, and pathological features (histologic subtype, lymph node status, tumor size, and other pathological features), were extracted from the HUH electronic records and patient files. Although patients were originally staged according to the 7th edition of the Tumor Node Metastasis (TNM) classification at the time of diagnosis, all cases were retrospectively reclassified according to the 8th edition for the purposes of the present analysis. Restaging was performed based on information obtained from the original pathology reports and institutional clinical records. Only minimal stage migration was observed after reclassification, and the overall stage distribution remained largely unchanged. Treatment-related variables (type of surgery, receipt of neoadjuvant and/or adjuvant therapy, radiotherapy, recurrence status, and relevant dates) were recorded.

During the study period (2009–2016), adjuvant chemotherapy was most commonly based on platinum-containing regimens, predominantly cisplatin plus vinorelbine. In the neoadjuvant setting, the most frequently administered regimens included gemcitabine plus cisplatin or gemcitabine plus cisplatin combined with paclitaxel, with regimen selection generally guided by histological subtype and institutional treatment practices. Radiotherapy was administered when clinically indicated according to standard treatment approaches during the study period.

### 2.3. Tissue Microarray Construction and Immunohistochemistry

Formalin-fixed, paraffin-embedded (FFPE) tumor tissue blocks were retrieved from the archives of the Department of Pathology at HUH. All available hematoxylin–eosin–stained slides were reviewed, and representative tumor areas were selected for tissue microarray (TMA) construction. Using a manual tissue microarray punch, two 4 mm cores were obtained from each selected tumor area and embedded into recipient paraffin blocks. Both cores were obtained from the same FFPE tumor block. Representative tumor regions were selected by an experienced pathologist while avoiding necrotic or non-tumoral areas.

From each TMA block, serial sections of 5 µm thickness were cut. One section was stained with hematoxylin–eosin for histopathological confirmation, and additional sections were used for immunohistochemical analysis. Sections were incubated at 60 °C for 30 min, then deparaffinized in xylene and rehydrated through graded ethanol solutions.

Endogenous peroxidase activity was blocked using 3% hydrogen peroxide. Antigen retrieval was performed with ER1 buffer (sodium citrate, pH 6.0) using an automated Leica Bond-Max™ immunostaining system, according to the manufacturer’s instructions. Slides were then incubated with a rabbit polyclonal anti-SULF2 antibody (LifeSpan BioSciences, Seattle, WA, USA, Anti-SULF2/Sulfatase-2 Antibody, aa 337–654, LS-C424339) at a 1:250 dilution, followed by the Novolink™ Polymer anti-rabbit Poly-HRP-IgG secondary antibody.

Immunoreactivity was visualized using 3,3′-diaminobenzidine chromogen, and slides were counterstained with hematoxylin. Appropriate positive and negative controls were included in each staining run. Based on Human Protein Atlas data, bronchial epithelium and macrophages served as internal positive controls, salivary gland tissue was used as an external positive control, and adipose tissue and skeletal muscle were used as external negative controls. Consistent with previously reported expression patterns, SULF2 immunoreactivity was predominantly observed in the cytoplasm of tumor cells.

### 2.4. Evaluation of Immunohistochemical SULF2 Expression

All immunohistochemically stained slides were independently evaluated by two experienced pathologists, one of whom specialized in thoracic pathology, and both were blinded to the patients’ clinical data and survival outcomes. The evaluation focused on both the extent and intensity of SULF2 staining, and the H-score was calculated as the product of these two parameters. Tumoral and stromal staining were not evaluated separately; overall immunohistochemical staining was assessed, consistent with prior studies using similar methodology.

The extent of staining was graded as follows: 0, no staining; 1, staining in 1–25% of tumor cells; 2, staining in 26–50%; 3, staining in 51–75%; and 4, staining in 76–100% of tumor cells. Staining intensity was scored as 0 (no staining), 1 (weak), 2 (moderate), and 3 (strong). Overall, interobserver agreement was high, and any discrepancies were resolved by consensus review. The resulting H-score ranged from 0 to 12. The raw distribution of these categories is presented in Table 2.

To facilitate statistical analyses and ensure adequate statistical power, SULF2 expression was categorized as low or high according to expression extent, staining intensity, and H-score. Expression extent was defined as low when staining involved ≤50% of tumor cells and high when >50% of tumor cells were positive. Staining intensity was classified as low in cases with negative or weak staining and high in cases with moderate or strong staining.

Accordingly, tumors with an H-score of 6–12 were classified as SULF2-high, corresponding to tumors showing >50% SULF2-positive cells with moderate or strong staining intensity. Tumors with H-scores of 0–4 were classified as SULF2-low, reflecting cases with ≤50% positive tumor cells and/or weak or absent staining intensity. This approach aligns with previously reported immunohistochemical studies, where biologically meaningful thresholds based on staining extent and intensity are preferred over purely statistical cut-offs, with high SULF2 expression defined by widespread tumor cell positivity together with moderate-to-strong staining intensity [19].

### 2.5. Outcomes of the Study

The primary outcome of the study was to evaluate the association between SULF2 expression and overall survival (OS). OS was defined as the interval from the date of diagnosis to death from any cause. Patients who were alive at the last follow-up were censored on that date. Because a proportion of patients received neoadjuvant therapy prior to surgery, OS was anchored to the date of diagnosis to capture the entire treatment course.

Secondary outcomes included the evaluation of the association between SULF2 expression and disease-free survival (DFS), as well as the assessment of the relationship between SULF2 expression and clinicopathological characteristics to explore its prognostic significance. DFS was defined as the interval from the date of curative-intent surgery to the first documented recurrence (local or distant) or death from any cause, whichever occurred first. Patients without recurrence or death were censored at the date of last disease assessment or last follow-up. Vital status was verified through the Turkish Ministry of Health Death Notification System.

### 2.6. Statistical Analysis

Descriptive statistics were used to summarize patient characteristics. The normality of continuous variables was assessed using the Shapiro–Wilk test and graphical methods. Continuous variables were expressed as mean ± standard deviation or median (interquartile range), as appropriate, and categorical variables as counts and percentages. Between-group comparisons were performed using the chi-square or Fisher’s exact test for categorical variables and Student’s *t*-test or Mann–Whitney U test for continuous variables, depending on the distribution of the data.

Survival outcomes included OS and DFS, as defined above. Survival curves were estimated using the Kaplan–Meier method and compared using the log-rank test.

Variables with established clinical relevance and/or a *p*-value < 0.25 in univariate analyses were considered for inclusion in the multivariate models. Multivariate survival analyses were performed using the Cox proportional hazards regression model (enter method), and results were reported as hazard ratios (HRs) with 95% confidence intervals (CIs). To avoid collinearity and model overfitting, variables with strong intercorrelations were not entered simultaneously into the multivariate models. A two-sided *p* value < 0.05 was considered statistically significant. All statistical analyses were performed using IBM SPSS Statistics, version 31.0.

## 3. Results

### 3.1. Patient Characteristics

A total of 83 patients with stage I–III NSCLC who underwent curative-intent surgical resection were included in the study. The median age was 62.1 years (IQR: 57.5–66.2), and the majority of patients were male (78.3%). Most patients had a good performance status, with 83.1% having an ECOG performance status of 0.

According to the TNM 8th edition staging system, 42.2% of patients had stage I disease, 32.5% had stage II disease, and 25.3% had stage III disease. Lobectomy was the most frequently performed surgical procedure (81.9%), whereas 18.1% of patients underwent pneumonectomy. Among patients who underwent pneumonectomy (*n* = 15), most had more advanced disease, including 8 patients with stage III and 5 with stage II disease, whereas only 2 patients had stage I tumors. Neoadjuvant treatment was administered to 14.5% of patients, and 38.6% received adjuvant therapy. Detailed demographic and clinicopathological characteristics of the study population are summarized in Table 3.

### 3.2. SULF2 Expression Evaluation

**Figure 2:**
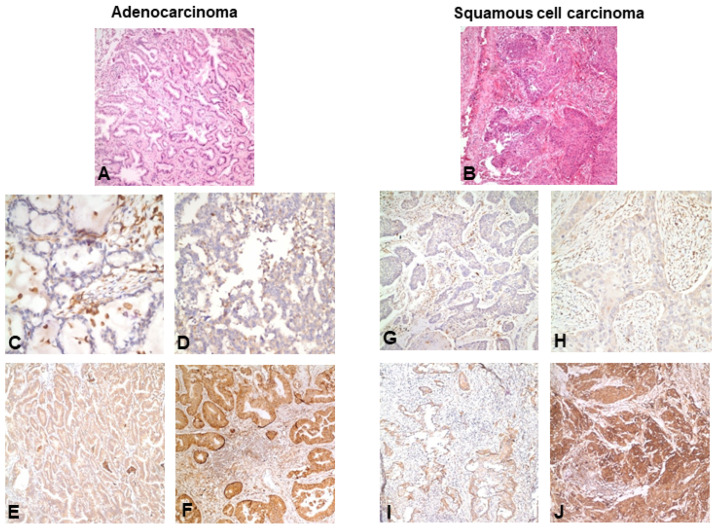
**Immunohistochemical Expression of SULF2 in Resected Non-Small Cell Lung Cancer.** Representative hematoxylin and eosin (H&E)–stained sections of lung adenocarcinoma (**A**) and squamous cell carcinoma (**B**), and immunohistochemical images illustrating SULF2 expression patterns in resected non-small cell lung cancer. (**C**–**F**) Representative adenocarcinoma specimens showing negative (**C**), weak (**D**), moderate (**E**), and strong (**F**) cytoplasmic SULF2 immunoreactivity. (**G**–**J**) Representative squamous cell carcinoma specimens showing negative (**G**), weak (**H**), moderate (**I**), and strong (**J**) cytoplasmic SULF2 immunoreactivity. SULF2 expression was predominantly localized to the cytoplasm of tumor cells. All images are shown at the same magnification.

SULF2 immunohistochemical expression was successfully evaluated in all tumor specimens included in the study. SULF2 immunoreactivity of any degree was detected in 78 patients (94.0%), whereas 5 patients (6.0%) had no detectable SULF2 expression. When stratified by histologic subtype, SULF2 positivity was observed in 39 of 40 AC (97.5%) and in 39 of 43 SCC (90.7%), indicating a high prevalence of SULF2 expression across both major NSCLC histological subtypes. Representative immunohistochemical staining patterns of SULF2 in AC and SCC specimens, including negative, weak, moderate, and strong expression levels, are shown in Figure 2.

According to expression extent, the majority of tumors demonstrated widespread SULF2 positivity, with 65 patients (78.3%) showing SULF2 expression in more than 50% of tumor cells. In contrast, 13 patients (15.7%) had SULF2 expression in ≤50% of tumor cells and 5 patients (6.0%) were negative. With respect to staining intensity, moderate or strong SULF2 expression was observed in 45 patients (54.2%), while 38 patients (45.8%) exhibited negative or weak staining.

Based on H-score dichotomization (0–4 vs. 6–12), 40 patients (48.2%) were classified as having low SULF2 expression and 43 patients (51.8%) as having high SULF2 expression. The detailed distribution of SULF2 staining extent, intensity, and H-score categories is presented in Table 4.

Comparisons of clinicopathological characteristics according to SULF2 H-score-based expression groups (SULF2-low [0–4] vs. SULF2-high [6–12]) are presented in Table 5. Patients in the SULF2-high group more frequently had ECOG performance status 1 (*p* = 0.028) and anemia (*p* = 0.008). No statistically significant differences were observed between the SULF2-low and SULF2-high groups with respect to age, sex, smoking status, histological subtype, pathological stage, surgical procedure, or receipt of neoadjuvant or adjuvant treatment.

### 3.3. Survival Outcomes

The median follow-up time was 91 months, calculated using the reverse Kaplan–Meier method. At the time of analysis, the median OS for the entire cohort was 113.2 months (95% CI: 39.4–186.9), and the median DFS was 85.2 months (95% CI: 44.6–125.7).

Overall survival differed significantly according to pathological stage (log-rank *p* = 0.029). Median OS was not reached in patients with stage I disease, whereas median OS was 54.9 months (95% CI: 44.6–65.2) for patients with stage II disease and 59.9 months (95% CI: 19.0–100.8) for those with stage III disease. Disease-free survival also differed significantly according to pathological stage (log-rank *p* < 0.001). Median DFS was not reached in patients with stage I disease, while median DFS was 49.4 months (95% CI: 41.3–57.5) for stage II disease and 20.5 months (95% CI: 5.7–35.2) for stage III disease.

**Figure 3:**
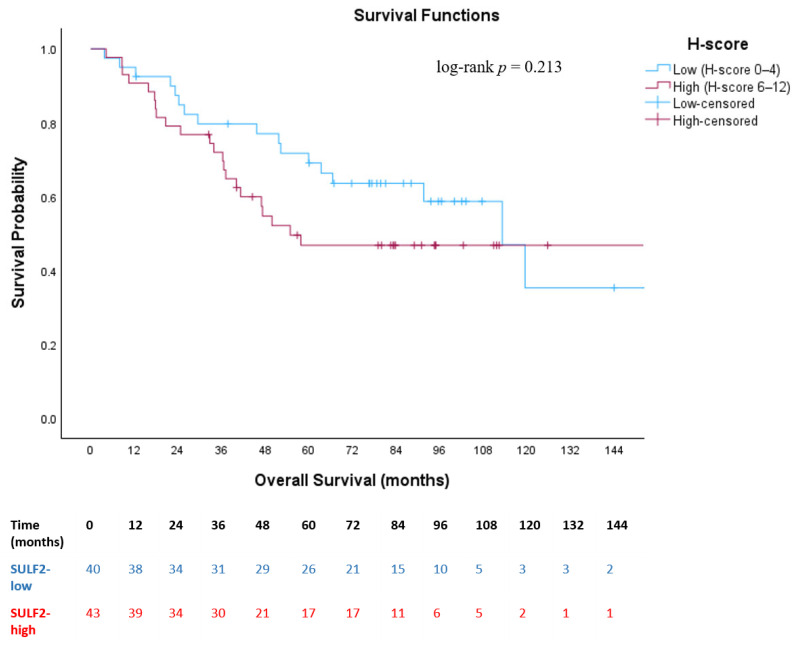
**Kaplan–Meier curve for overall survival according to SULF2 expression status (SULF2-low [H-score 0–4] vs. SULF2-high [H-score 6–12]).** Kaplan–Meier curves illustrating overall survival stratified by SULF2 expression based on H-score categories (low vs. high) in patients with resected stage I–III non-small cell lung cancer. Tick marks indicate censored patients.

**Figure 4:**
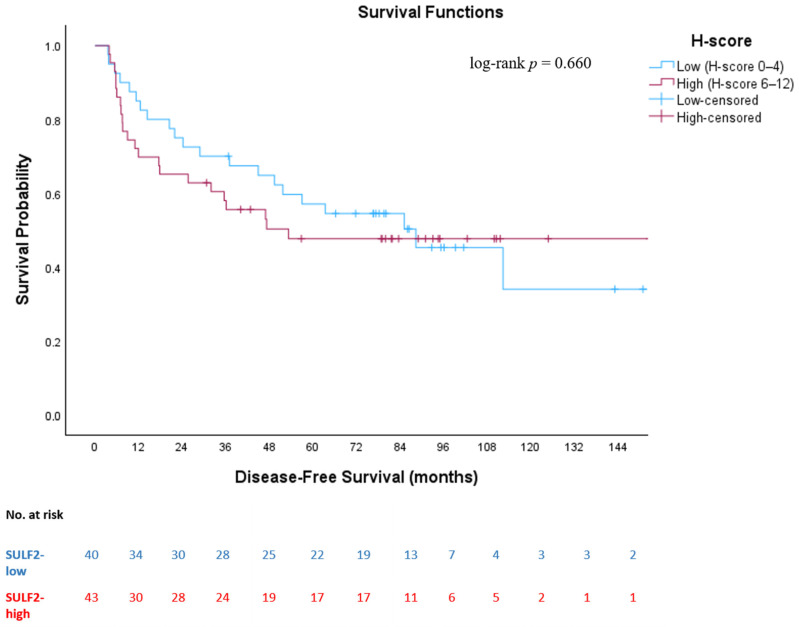
**Kaplan–Meier curve for disease-free survival according to SULF2 expression status (SULF2-low [H-score 0–4] vs. SULF2-high [H-score 6–12]).** Kaplan–Meier curves illustrating disease-free survival stratified by SULF2 expression based on H-score categories (low vs. high) in patients with resected stage I–III non-small cell lung cancer. Tick marks indicate censored patients.

When stratified according to SULF2 H-score-based expression groups, no statistically significant difference in OS was observed between patients with low and high SULF2 expression (log-rank *p* = 0.213). Median OS was 113.2 months (95% CI: 84.0–142.4) in the SULF2-low group and 54.9 months (95% CI: 39.4–186.9) in the SULF2-high group (Figure 3). Similarly, DFS did not differ statistically significantly between SULF2-low and SULF2-high groups (log-rank *p* = 0.660). Median DFS was 88.3 months (95% CI: 46.5–130.2) in the SULF2-low group and 53.3 months in the SULF2-high group (Figure 4). During follow-up, a total of 39 OS events (deaths) and 43 DFS events (recurrence or death) were recorded in the overall cohort. When stratified by SULF2 expression, OS events occurred in 17 patients in the SULF2-low group and 22 patients in the SULF2-high group, while DFS events were observed in 21 and 22 patients, respectively.

Subgroup analyses stratified by pathological stage demonstrated no statistically significant differences in OS or DFS between low and high SULF2 expression groups within each pathological stage (OS: log-rank *p* = 0.151; DFS: log-rank *p* = 0.518). Kaplan–Meier curves showed largely overlapping survival patterns across all pathological stages (Supplementary Figures S1–S6).

Additional analyses evaluating SULF2 expression extent (≤50% vs. >50%) did not demonstrate statistically significant differences in OS or DFS (OS: log-rank *p* = 0.381; DFS: log-rank *p* = 0.381). Similarly, analyses based on expression intensity (negative/weak vs. moderate/strong) did not demonstrate statistically significant differences in OS or DFS (OS: log-rank *p* = 0.187; DFS: log-rank *p* = 0.665).

Furthermore, histology-specific subgroup survival analyses were performed. In histology-stratified Cox regression analyses, SULF2 expression was not significantly associated with OS or DFS in either AC or SCC subgroups. In AC patients, SULF2-high expression was associated with a lower hazard of death compared with SULF2-low expression (HR 0.50, *p* = 0.197), although this did not reach statistical significance, and showed no association with DFS (HR 1.04, *p* = 0.928). In SCC patients, SULF2-high expression was not significantly associated with OS (HR 0.69, *p* = 0.390) or DFS (HR 1.33, *p* = 0.502).

### 3.4. Cox Regression Analyses for Overall and Disease-Free Survival

In univariate Cox regression analysis for OS, age ≥ 65 years, ECOG performance status 1, higher Charlson Comorbidity Index (CCI), advanced pathological stage, pneumonectomy, and receipt of neoadjuvant or adjuvant chemotherapy were significantly associated with worse OS (Table 6). However, SULF2 expression status based on H-score (low vs. high) was not significantly associated with OS in univariate analysis.

Variables that met the predefined criteria in univariate analyses and were considered clinically relevant were included in the multivariate Cox regression model. To avoid collinearity and overfitting, CCI and treatment-related variables (neoadjuvant and adjuvant chemotherapy) were excluded because of their strong correlations with age, pathological stage, and surgical procedure. In multivariate analysis, surgical procedure remained independent prognostic factor for OS. Patients who underwent pneumonectomy had a higher risk of mortality compared with those who underwent lobectomy (HR 2.15, 95% CI 1.00–4.63, *p* = 0.050). Age, ECOG performance status, pathological stage, and SULF2 H-score were not independently associated with OS in the multivariate model (Table 6).

For DFS, univariate Cox regression analysis demonstrated that older age, higher comorbidity burden, advanced pathological stage, pneumonectomy, and receipt of neoadjuvant or adjuvant chemotherapy were significantly associated with worse DFS (Table 7). SULF2 expression based on H-score was not associated with DFS in univariate analysis. Multivariate Cox regression analysis showed that pathological stage and surgical procedure were independent predictors of DFS. Patients with stage III disease had a higher risk of recurrence or death compared with those with stage I disease (HR 2.61, 95% CI 1.10–6.21, *p* = 0.030). In addition, pneumonectomy was independently associated with worse DFS compared with lobectomy (HR 2.14, 95% CI 1.03–4.43, *p* = 0.041). SULF2 H-score did not show an independent association with DFS in the multivariate model.

## 4. Discussion

In this study, we comprehensively evaluated the prognostic significance of SULF2 expression in surgically resected stage I–III NSCLC using immunohistochemical analysis. With a long median follow-up of 91 months, patients with high SULF2 expression showed numerically shorter OS and DFS; however, these differences did not reach statistical significance. Similar patterns were observed in stage-stratified analyses, and no significant differences were identified between AC and SCC subgroups. Taken together, these findings indicate a numerical trend toward poorer prognosis in patients with high SULF2 expression. The lack of statistical significance is likely attributable to the limited sample size and number of events. Importantly, our study provides long-term outcome data from a homogeneous cohort of surgically treated stage I–III NSCLC patients, and contributes to clarifying the prognostic role of SULF2 expression in early-stage disease, where current evidence remains limited and inconsistent.

SULF2 is an extracellular endosulfatase that regulates the sulfation pattern of heparan sulfate proteoglycans, thereby modulating the bioavailability and signaling activity of multiple growth factors and morphogens, including *Wnt*, *FGF*, *VEGF*, and *TGF-β* [6]. Through these mechanisms, SULF2 has been implicated in several key oncogenic processes, including tumor initiation, invasion, epithelial–mesenchymal transition, and resistance to therapy [6,8]. Experimental studies in lung cancer models have consistently shown that SULF2 overexpression promotes malignant transformation, enhances invasive capacity, and facilitates tumor growth, whereas genetic or epigenetic silencing of SULF2 reverses these aggressive phenotypes [20]. These findings suggest that increased SULF2 expression may be associated with more aggressive tumor behavior and potentially poorer clinical outcomes, providing a biological rationale for investigating its prognostic relevance in NSCLC.

One of the first studies evaluating SULF2 expression in lung cancer reported higher expression levels in SCC compared with AC [20]. A subsequent study evaluating SULF2 as a diagnostic and prognostic biomarker reported SULF2 immunohistochemical staining in most lung cancer specimens, with higher staining levels observed in SCC than in AC (100% vs. 60%; *p* < 0.0005) [21]. In our cohort, SULF2 expression was detected in 78 of 83 patients (94.0%). When stratified by histological subtype, SULF2 positivity was observed in 39 of 40 AC (97.5%) and in 39 of 43 SCC (90.7%). In contrast to previous reports suggesting higher expression predominantly in SCC, our findings indicate a broadly comparable distribution of SULF2 expression across both major NSCLC histological subtypes. In our study, SULF2 expression was categorized using biologically meaningful thresholds that incorporated both staining extent and intensity, consistent with prior immunohistochemical studies, and the results remained stable across different expression levels [19].

The prognostic role of SULF2 expression in surgically resected NSCLC has been previously investigated by Lui et al., who reported a histology-dependent association between SULF2 expression and survival outcomes [21]. In that study, SULF2 staining in tumor cells was associated with a non-significant 31% increase in the risk of death in patients with AC (*p* = 0.65), whereas 89% reduction in mortality risk was observed in patients with SCC (*p* = 0.02). These findings, however, were derived from relatively small, histology-specific subgroups and were characterized by a markedly imbalanced distribution of SULF2-negative cases, particularly within the SCC cohort. In contrast, histology-specific analyses in our cohort did not demonstrate a significant association between SULF2 expression and survival outcomes in either AC or SCC. These observations suggest that previously reported histology-dependent prognostic effects should be interpreted with caution, and that our findings do not exclude such an effect but rather indicate that it could not be confirmed with sufficient precision, particularly in the context of limited subgroup sizes.

In our study, median OS was 113.2 months (95% CI: 84.0–142.4) in the SULF2-low group and 54.9 months (95% CI: 39.4–186.9) in the SULF2-high group (log-rank *p* = 0.213), while median DFS was 88.3 months (95% CI: 46.5–130.2) and 53.3 months, respectively (log-rank *p* = 0.660). Although patients with high SULF2 expression showed numerically shorter survival outcomes, suggesting a potential adverse prognostic effect, this difference did not reach statistical significance. This finding may be attributable to the limited number of events and wide confidence intervals, which reduce the statistical power to detect a modest prognostic effect.

Similarly, several variables that appeared significant in univariate analyses did not remain significant in the multivariate model. This may reflect shared variance among clinicopathological variables and the limited number of outcome events, both of which can affect the stability of multivariable estimates in relatively small cohorts. The observed association between pneumonectomy and survival outcomes should also be interpreted with caution, as the extent of surgical resection is closely related to tumor burden, anatomical complexity, and operability, and may therefore reflect underlying disease severity rather than an independent prognostic effect. Furthermore, the associations observed for neoadjuvant and adjuvant treatments in univariate analyses are likely influenced by confounding by indication, as patients with more advanced or aggressive disease are more likely to receive systemic therapy; therefore, these findings should not be interpreted as causal treatment effects. Collectively, these factors may have further attenuated the detectable prognostic impact of SULF2 expression in multivariable analyses.

SULF2 expression has been associated with disease severity in other solid tumors, such as hepatocellular carcinoma, where high expression in resected tumors correlates with aggressive features and poorer survival [15]. In our cohort, although not statistically significant, high SULF2 expression showed numerically poorer survival outcomes and was more frequently observed in patients with adverse clinical characteristics, including poorer performance status and anemia. These effects may be more relevant in advanced disease settings, whereas in early-stage or completely resected NSCLC they may be attenuated by effective surgical clearance and multimodal treatment and therefore may not be fully captured in our study.

The biological effects of SULF2 appear to be highly context dependent and influenced by tumor–microenvironment interactions rather than tumor cell–intrinsic expression alone [6,8]. Moreover, SULF2 expression is not restricted to tumor cells but is also observed in stromal components, including fibroblasts and inflammatory cells, suggesting that its prognostic relevance may depend on complex tumor–stroma crosstalk [15,23,24]. However, in the present study, SULF2 expression was evaluated as a combined immunohistochemical signal without separate assessment of tumor and stromal compartments, which may have diluted potential associations with survival outcomes. In addition, conventional immunohistochemical assessment may not fully capture this spatial and functional heterogeneity or reflect enzymatic activity, post-translational regulation, or epigenetic modulation of SULF2. In this context, epigenetic silencing of SULF2 through promoter methylation has been associated with improved survival and increased sensitivity to topoisomerase-I inhibitors, highlighting the complexity of its biological regulation [25].

The present study focused specifically on immunohistochemical assessment of tissue-level SULF2 protein expression; therefore, transcriptomic analyses and comprehensive molecular or epigenetic evaluations were beyond the scope of this retrospective design. Importantly, the focus on immunohistochemical protein expression reflects a clinically applicable approach, as IHC remains the most widely used method for biomarker evaluation in routine pathological practice. Accordingly, our study provides clinically translatable data based on a widely accessible methodology; however, integration of transcriptomic, circulating, and epigenetic data would allow a more comprehensive characterization of SULF2 biology. Such multi-layered analyses were beyond the predefined scope of this retrospective, tissue-based study and require prospectively collected biospecimens and dedicated molecular platforms.

Evidence from other malignancies further supports a biologically relevant role for SULF2 in cancer progression [8]. An analysis of the Oncomine public gene microarray database reported upregulated SULF2 expression in malignant brain tumors, breast cancer, head and neck cancers, and papillary renal cell carcinoma, further supporting a role for SULF2 dysregulation across multiple malignancies [6]. In breast cancer, SULF2 has been shown to promote cell proliferation, invasion, and migration, while inhibiting apoptosis, as demonstrated in both in vitro and in vivo studies [24]. In addition, studies evaluating SULF2 expression in hepatocellular carcinoma, HNSCC, pancreatic ductal adenocarcinoma, bladder cancer, and ACC have reported associations between elevated SULF2 levels and more aggressive tumor behavior or poorer clinical outcomes [15,16,17,18,19]. Notably, these studies have employed heterogeneous methodological approaches, including transcriptomic analyses, immunohistochemical protein assessment, and functional experimental models. Differences in scoring systems and the tissue compartment evaluated (tumor cells vs. stromal components) may also contribute to variability in reported clinical associations. Together, these findings highlight the context-dependent role of SULF2 in cancer biology and support its biological relevance beyond NSCLC.

Emerging evidence suggests that circulating SULF2 may serve as a potential blood-based biomarker for the early detection of lung cancer [21]. Plasma SULF2 levels have been reported to be significantly elevated in patients with NSCLC compared with healthy controls, including those with early-stage disease, although circulating SULF2 concentrations appear to increase with age [21,22]. Notably, a tumor-specific SULF2 splice variant has been identified in the plasma of NSCLC patients but not in individuals with COPD or healthy controls, supporting the potential diagnostic specificity of circulating SULF2-based assays for early lung cancer detection [22]. However, circulating SULF2 levels were not evaluated in the present study because plasma samples were not prospectively collected during the study period, limiting retrospective assessment. Future studies integrating tissue-based SULF2 expression with circulating biomarkers and molecular profiling may help to better define the diagnostic, prognostic, and potentially predictive role of SULF2 in NSCLC.

Several limitations of this study should be acknowledged. First, the retrospective and single-center design may introduce inherent selection bias, despite the use of well-defined inclusion criteria and comprehensive clinicopathological data. Second, the relatively limited sample size may have reduced the statistical power to detect modest survival effects associated with SULF2 expression. Expansion of the cohort was not feasible due to limitations in tissue availability and completeness of long-term follow-up data. Third, SULF2 assessment was based on immunohistochemical evaluation of archival FFPE tissue, which may not fully reflect functional enzymatic activity, epigenetic regulation, or temporal changes in expression during disease progression. The absence of mRNA expression analysis, circulating SULF2 measurements, and epigenetic profiling represents an additional limitation, as these complementary approaches may provide further biological and translational insights. Moreover, intratumoral heterogeneity and the use of tissue microarray cores may have influenced classification into expression categories, as limited sampling may not fully capture the spatial variability of SULF2 expression across tumor and stromal compartments. Although all cases were reviewed by two experienced pathologists and discrepancies were resolved by consensus, formal assessment of interobserver reproducibility was not performed and represents an additional limitation. Finally, as our cohort was restricted to surgically resected stage I–III NSCLC patients, the findings may not be generalizable to advanced or unresectable disease. In this context, our results should be interpreted as hypothesis-generating and warrant further validation.

## 5. Conclusions

In conclusion, SULF2 expression was highly prevalent in patients with surgically resected stage I–III NSCLC; however, in our study, it could not be demonstrated as an independent prognostic biomarker. Although high SULF2 expression showed numerically shorter survival and was more frequently observed in patients with adverse clinical features, these findings were not statistically significant and were not consistently confirmed across stage- or histology-specific analyses. Nevertheless, given its biological relevance in lung cancer and the observed numerical differences in outcomes in the high-expression group, SULF2 may still warrant further investigation within integrated biomarker models. Future prospective studies incorporating larger cohorts and combined tissue-based, circulating, and molecular analyses are needed to better define the clinical relevance of SULF2 in NSCLC.

## Figures and Tables

**Table 1:** Inclusion and exclusion criteria for the study cohort.

Inclusion Criteria	Exclusion Criteria
Histologically confirmed NSCLC (adenocarcinoma or squamous cell carcinoma)	Small cell lung cancer, large cell lung cancer, neuroendocrine carcinoma
Surgically resected NSCLC	Non-operated or inoperable disease (stage IV or unresectable)
Pathological stage I–III disease, (TNM 8th edition)	History of another malignancy
Availability of archived FFPE tumor tissue	No available tumor tissue
Adequate tissue for SULF2 immunohistochemistry	Insufficient tissue for immunohistochemical analysis
Availability of complete clinical and follow-up data *	Incomplete or inaccessible clinical data

* Complete clinical data included demographic characteristics, pathological stage, treatment information, and survival status. Abbreviations: NSCLC, non–small cell lung cancer; TNM, tumor–node–metastasis; FFPE, formalin-fixed paraffin-embedded.

**Table 2:** Distribution of patients according to SULF2 expression extent, intensity, and H-score.

Characteristic	Total (*n* = 83), *n* (%)
Extent of Expression	
0%	5 (6.0)
1–25%	6 (7.2)
26–50%	7 (8.4)
51–75%	7 (8.4)
76–100%	58 (69.9)
Intensity of Expression	
Negative	5(6.0)
Weak	33 (39.8)
Moderate	28 (33.7)
Strong	17 (20.5)
H-score	
Score 0–3	20 (24.1)
Score 4–6	23 (27.7)
Score 8–12	40 (48.2)

This table presents the raw distribution of SULF2 expression according to staining extent, intensity, and H-score prior to regrouping. Abbreviations: SULF2, sulfatase 2.

**Table 3:** Baseline Clinicopathological Characteristics of the Study Cohort.

Characteristic	Total (*n* = 83)
Age, years, median (IQR)	62.1 (57.5–66.2)
Gender	
Female	18 (21.7%)
Male	65 (78.3%)
Smoking status	
Non-smoker	13 (15.7%)
Ever-smoker (current/former)	62 (74.7%)
Unknown	8 (9.6%)
ECOG performance status	
ECOG 0	69 (83.1%)
ECOG 1	14 (16.9%)
Charlson Comorbidity Index (CCI)	
≤5	43 (51.8%)
>5	40 (48.2%)
Tumor location	
Central	40 (48.2%)
Peripheral	37 (44.6%)
Superior sulcus	6 (7.2%)
Tumor laterality	
Left lung	36 (43.4%)
Right lung	47 (56.6%)
Histology	
Adenocarcinoma	40 (48.2%)
Squamous cell carcinoma	43 (51.8%)
Pathologic Stage (AJCC 8th edition)	
Stage I	35 (42.2%)
Stage II	27 (32.5%)
Stage III	21 (25.3%)
Surgery type	
Lobectomy	68 (81.9%)
Pneumonectomy	15 (18.1%)
Neoadjuvant treatment	
Present	12 (14.5%)
Absent	71 (85.5%)
Adjuvant treatment	
Present	32 (38.6%)
Absent	51 (61.4%)

Abbreviations: IQR, interquartile range; ECOG, Eastern Cooperative Oncology Group; CCI, Charlson Comorbidity Index; AJCC, American Joint Committee on Cancer.

**Table 4:** Definition of SULF2-low and SULF2-high expression groups based on expression extent, intensity, and H-score *.

Characteristic	SULF2-Low *n* (%)	SULF2-High *n* (%)
Extent of Expression (≤50% vs. >50%)	18 (21.7%)	65 (78.3%)
Intensity of Expression (negative/weak vs. moderate/strong)	38 (45.8%)	45 (54.2%)
H-score category (0–4 vs. 6–12)	40 (48.2%)	43 (51.8%)

* SULF2-low and SULF2-high expression groups were defined according to predefined cut-off values for expression extent, staining intensity, and H-score, as described in Section 2 (Materials and Methods). Abbreviations: SULF2, sulfatase 2.

**Table 5:** Comparison of Clinicopathological Characteristics Between SULF2-Low and SULF2-High Expression Groups.

Characteristic	SULF2 Low *n* = 40 (%)	SULF2 High *n* = 43 (%)	*p*-Value
Age, years, median (IQR)	61.7 (57.8–64.6)	62.5 (57.0–67.5)	0.639
Gender			0.862
Female	9 (22.5%)	9 (20.9%)	
Male	31 (77.5%)	34 (79.1%)	
Smoking status *			0.283
Never-smoker	8 (22.2%)	5 (12.8%)	
Ever-smoker (current/former)	28 (77.8%)	34 (87.2%)	
ECOG performance status			0.028
ECOG 0	37 (92.5%)	32 (74.4%)	
ECOG 1	3 (7.5%)	11 (25.6%)	
Charlson Comorbidity Index			0.150
≤5	24 (60.0%)	19 (44.2%)	
>5	16 (40.0%)	24 (55.8%)	
Tumor location			0.751
Central	20 (50.0%)	20 (46.5%)	
Peripheral	20 (50.0%)	23 (53.5%)	
Histology			0.751
Adenocarcinoma	20 (50.0%)	20 (46.5%)	
Squamous cell carcinoma	20 (50.0%)	23 (53.5%)	
Pathologic Stage (AJCC 8th edition)			0.634
Stage I	16 (40.0%)	19 (44.2%)	
Stage II	12 (30.0%)	15 (34.9%)	
Stage III	12 (30.0%)	9 (20.9%)	
Surgery type			0.114
Lobectomy	30 (75.0%)	38 (88.4%)	
Pneumonectomy	10 (25.0%)	5 (11.6%)	
Neoadjuvant treatment			0.447
Present	7 (17.5%)	5 (11.6%)	
Absent	33 (82.5%)	38 (88.4%)	
Adjuvant treatment			0.521
Present	14 (35.0%)	18 (41.9%)	
Absent	26 (65.0%)	25 (58.1%)	
Anemia			0.008
Present	19 (48.7%)	9 (20.9%)	
Absent	20 (51.3%)	34 (79.1%)	

* Patients with missing smoking data were excluded from this comparison. Abbreviations: IQR, interquartile range; ECOG, Eastern Cooperative Oncology Group; AJCC, American Joint Committee on Cancer.

**Table 6:** Univariate and Multivariate Cox Regression Analyses of Prognostic Factors for Overall Survival.

Parameter	Univariate Analysis		Multivariate Analysis	
	HR (95% CI)	*p*-Value	HR (95% CI)	*p*-Value
Age (<65 vs. ≥65 years)	2.51 (1.32–4.78)	0.005	1.66 (0.81–3.34)	0.168
ECOG (0 vs. 1)	2.23 (1.07–4.63)	0.032	1.73 (0.81–3.72)	0.159
Charlson Comorbidity Index (≤5 vs. >5)	2.60 (1.35–5.02)	0.004		
TNM Stage		0.037		0.252
Stage I vs. II	2.24 (1.01–5.01)	0.048	1.79 (0.78–4.09)	0.168
Stage I vs. III	2.82 (1.25–6.35)	0.013	2.01 (0.84–4.81)	0.118
Lobectomy vs. Pneumonectomy	2.79 (1.37–5.68)	0.005	2.15 (1.00–4.63)	0.050
SULF2 H score (Low vs. High)	1.50 (0.79–2.83)	0.216	1.56 (0.80–3.05)	0.196
Neoadjuvant chemotherapy (No vs. Yes)	2.27 (1.11–4.66)	0.026		
Adjuvant chemotherapy (No vs. Yes)	1.97 (1.03–3.76)	0.039		

Variables significant in univariate analysis were considered for multivariate modeling; Charlson Comorbidity Index and neoadjuvant/adjuvant chemotherapy were excluded due to collinearity with age, stage, ECOG performance status, and surgical procedure. Abbreviations: HR, hazard ratio; CI, confidence interval; ECOG, Eastern Cooperative Oncology Group; TNM, tumor–node–metastasis; SULF2, sulfatase 2.

**Table 7:** Univariate and Multivariate Cox Regression Analyses of Prognostic Factors for Disease-Free Survival.

Parameter	Univariate Analysis		Multivariate Analysis	
	HR (95% CI)	*p*-Value	HR (95% CI)	*p*-Value
Age (<65 vs. ≥65 years)	2.17 (1.17–4.02)	0.014	1.37 (0.70–2.68)	0.353
Female vs. Male	1.69 (0.75–3.81)	0.205	1.52 (0.63–3.64)	0.349
ECOG (0 vs. 1)	1.98 (0.97–4.05)	0.061	1.95 (0.89–4.28)	0.096
Charlson Comorbidity Index ≤5 vs. >5	2.37 (1.28–4.42)	0.006		
TNM Stage		0.002		0.069
Stage I vs. II	2.55 (1.16–5.63)	0.021	2.28 (1.0–4.97)	0.050
Stage I vs. III	4.12 (1.88–9.04)	<0.001	2.61 (1.10–6.21)	0.030
Lobectomy vs. Pneumonectomy	2.83 (1.46–5.47)	0.002	2.14 (1.03–4.43)	0.041
SULF2 H score (Low vs. High)	1.14 (0.63–2.08)	0.660	1.26 (0.68–2.36)	0.461
Neoadjuvant chemotherapy No vs. Yes	3.09 (1.51–6.34)	0.002		
Adjuvant chemotherapy No vs. Yes	2.29 (1.25–4.22)	0.008		

Variables significant in univariate analysis were considered for multivariate modeling; Charlson Comorbidity Index and neoadjuvant/adjuvant chemotherapy were excluded due to collinearity with age, stage, ECOG performance status, and surgical procedure. Abbreviations: HR, hazard ratio; CI, confidence interval; ECOG, Eastern Cooperative Oncology Group; TNM, tumor–node–metastasis; SULF2, sulfatase 2.

## Data Availability

The datasets analyzed in this study were derived from the author’s previously completed master’s thesis at Hacettepe University and are available from the corresponding author upon reasonable request.

## Supplementary Materials

The following supporting information can be downloaded at: https://www.mdpi.com/article/10.3390/medsci14020215/s1. Figure S1: Kaplan–Meier analysis of overall survival according to SULF2 H-score (low vs. high) in patients with pathologic stage I non-small cell lung cancer.; Figure S2: Kaplan–Meier analysis of overall survival according to SULF2 H-score (low vs. high) in patients with pathologic stage II non-small cell lung cancer.; Figure S3: Kaplan–Meier analysis of overall survival according to SULF2 H-score (low vs. high) in patients with pathologic stage III non-small cell lung cancer.; Figure S4: Kaplan–Meier analysis of disease-free survival according to SULF2 H-score (low vs. high) in patients with pathologic stage I non-small cell lung cancer.; Figure S5: Kaplan–Meier analysis of disease-free survival according to SULF2 H-score (low vs. high) in patients with pathologic stage II non-small cell lung cancer.; Figure S6: Kaplan–Meier analysis of disease-free survival according to SULF2 H-score (low vs. high) in patients with pathologic stage III non-small cell lung cancer.

## Abbreviations

The following abbreviations are used in this manuscript: AC: Adenocarcinoma; AJCC: American Joint Committee on Cancer; CI: Confidence Interval; DFS: Disease-Free Survival; HR: Hazard Ratio; IHC: Immunohistochemistry; IRB: Institutional Review Board; NSCLC: Non-Small Cell Lung Cancer; OS: Overall Survival; SCC: Squamous Cell Carcinoma; SULF2: Sulfatase 2; TMA: Tissue Microarray; TNM: Tumor–Node–Metastasis.
