# Supplementary material for: Prognostic Significance of SULF2 Expression in Surgically Resected Non-Small Cell Lung Cancer
Source: Med Sci (Basel). 2026 Apr 26;14(2):215. doi: 10.3390/medsci14020215 (PMC13214717; doi:10.3390/medsci14020215)
Supplement: Supplementary file 1 [file medsci-14-00215-s001.zip › Supplemantary Figure Legends.pdf]

Supplementary Figure Legends

Supplementary Figure S1. Kaplan–Meier analysis of overall survival according to SULF2 H-score (low vs. high) in patients with pathologic stage I non-small cell lung cancer.

Supplementary Figure S2. Kaplan–Meier analysis of overall survival according to SULF2 H-score (low vs. high) in patients with pathologic stage II non-small cell lung cancer.

Supplementary Figure S3. Kaplan–Meier analysis of overall survival according to SULF2 H-score (low vs. high) in patients with pathologic stage III non-small cell lung cancer.

Supplementary Figure S4. Kaplan–Meier analysis of disease-free survival according to SULF2 H-score (low vs. high) in patients with pathologic stage I non-small cell lung cancer.

Supplementary Figure S5. Kaplan–Meier analysis of disease-free survival according to SULF2 H-score (low vs. high) in patients with pathologic stage II non-small cell lung cancer.

Supplementary Figure S6. Kaplan–Meier analysis of disease-free survival according to SULF2 H-score (low vs. high) in patients with pathologic stage III non-small cell lung cancer.
