# Supplementary figures and images for: Prognostic Significance of SULF2 Expression in Surgically Resected Non-Small Cell Lung Cancer
Source: Med Sci (Basel). 2026 Apr 26;14(2):215. doi: 10.3390/medsci14020215 (PMC13214717; doi:10.3390/medsci14020215)

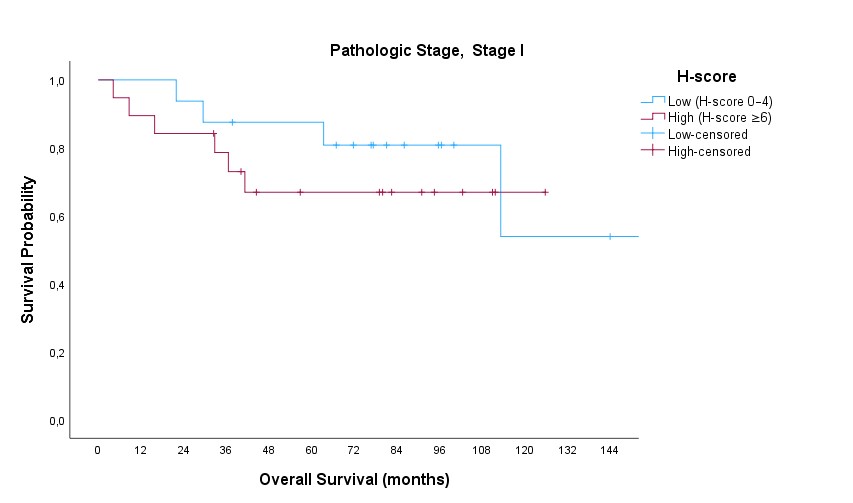

Supplement: Supplementary file 1 [file medsci-14-00215-s001.zip › Supplementary Figure S1.jpg]

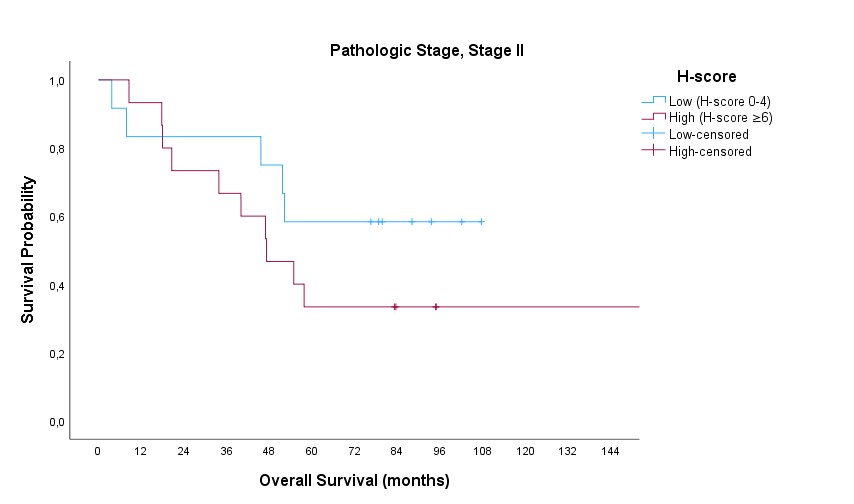

Supplement: Supplementary file 1 [file medsci-14-00215-s001.zip › Supplementary Figure S2.jpg]

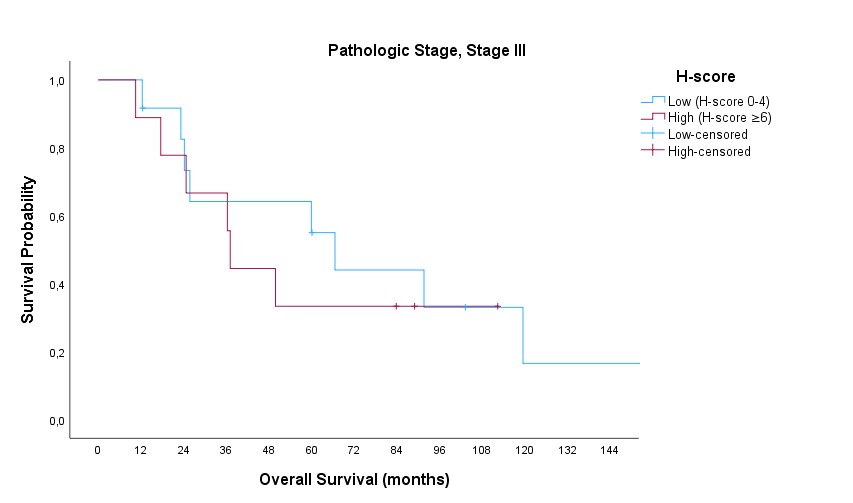

Supplement: Supplementary file 1 [file medsci-14-00215-s001.zip › Supplementary Figure S3.jpg]

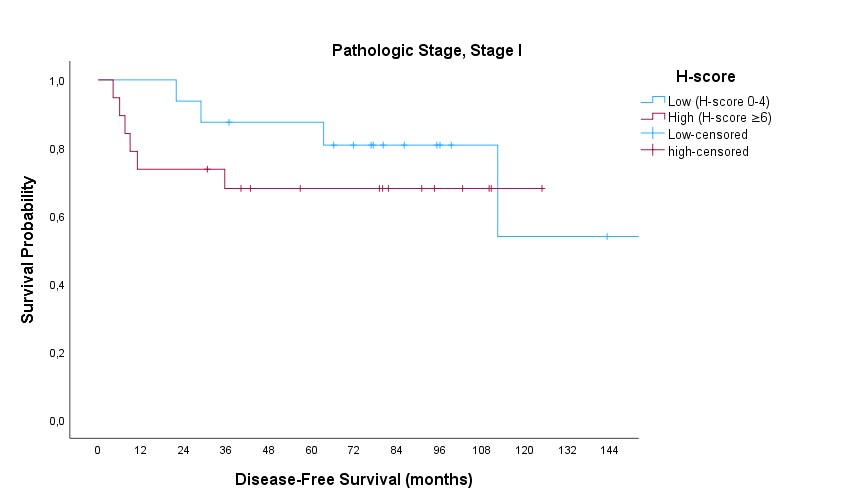

Supplement: Supplementary file 1 [file medsci-14-00215-s001.zip › Supplementary Figure S4.jpg]

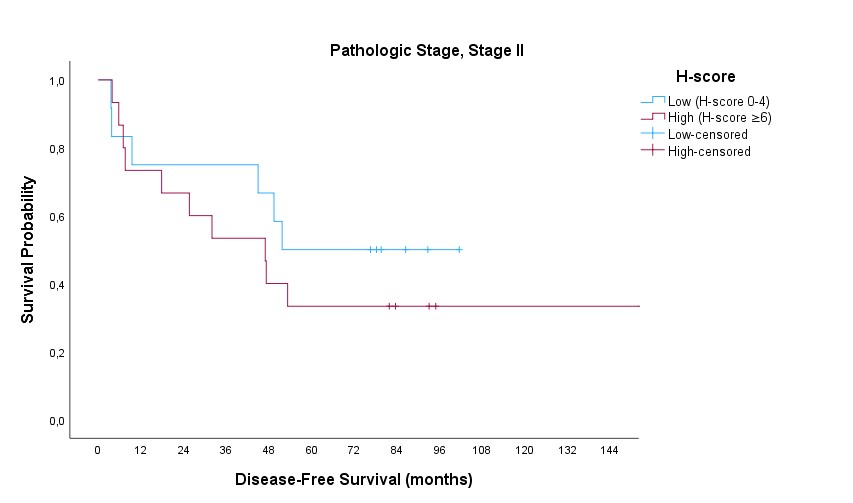

Supplement: Supplementary file 1 [file medsci-14-00215-s001.zip › Supplementary Figure S5.jpg]

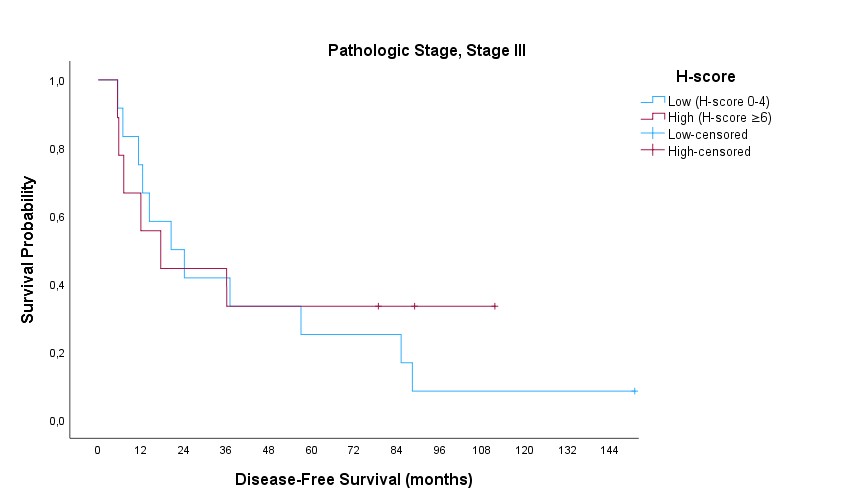

Supplement: Supplementary file 1 [file medsci-14-00215-s001.zip › Supplementary Figure S6.jpg]
